# Immunogenicities of vaccines including the immunoglobulin M-degrading enzyme of *Streptococcus suis*, rIde_*Ssuis*_, and protective efficacy against serotype 14 in piglets

**DOI:** 10.1016/j.jvacx.2024.100590

**Published:** 2024-11-21

**Authors:** L. Mayer, C. Liedel, K. Klose, A. de Greeff, K. Rieckmann, C.G. Baums

**Affiliations:** a Institute of Bacteriology and Mycology, Centre for Infectious Diseases, Veterinary Faculty, Leipzig University, Germany; b Institute of Veterinary Pathology, Faculty of Veterinary Medicine, Leipzig University, Leipzig, Germany; c Wageningen Bioveterinary Research, part of Wageningen University and Research, Lelystad,The Netherlands

**Keywords:** IgM, Pig vaccination, Cesarean-derived colostrum-deprived pigs, Arthritis, Meningitis

## Abstract

*Streptococcus suis* (*S. suis*) is a major porcine pathogen. Some strains have a substantial zoonotic potential such as serotype (*cps*) 14 as the second most important *cps* in human infections. To this date no licensed *S. suis* vaccine is available in Europe though subunit vaccines and bacterins have been examined by several scientific groups worldwide. Objectives of this study were to determine protective efficacy of rIde_*Ssuis*_ vaccination against intranasal *S. suis cps*14 challenge in conventional weaned piglets and to investigate additionally immunogenicity of rIde_*Ssuis*_ vaccination in cesarean-derived colostrum-deprived (CDCD) piglets. Immunization led to reduction of bacterial multiplicity in porcine blood and delayed onset of clinical signs of *cps*14 disease in conventional rIde_*Ssuis*_-vaccinated piglets. However, significant differences were not recorded which might be related to comparable low anti-Ide_*Ssuis*_ antibody levels and insufficient neutralization of IgM protease activity in this animal experiment. In contrast, immunization of cesarean-derived colostrum-deprived piglets with rIde_*Ssuis*_ resulted in high α-rIde_*Ssuis*_ IgG antibody levels and a highly significant reduction of the survival factor of the *cps*14 challenge strain in porcine blood *in vitro*. In conclusion, the results of this study indicate bactericidal immunity against *S. suis cps*14 by Ide_*Ssuis*_ specific immunity.

## 1 Introduction

*Streptococcus (S.) suis* is a worldwide distributed pathobiont causing severe diseases in pigs and humans [1]. Outbreaks in pig farms occur mainly in weaning piglets with clinical manifestations of arthritis, meningitis, serositis and septicemic shock. While *S. suis* covers at least 29 different serotypes (*cps)* differing in structure and composition of the capsule [2], few *cps* dominate in *S. suis* diseases. Worldwide *cps*2 and *cps*9 are most frequently isolated from diseased pigs. Strains of *cps*14 play an important role in the United Kingdom where herd problems due to *cps*14 usually occur at an age of 6–8 weeks of life [3]. Human *S. suis* infections are mainly caused by *cps*2, followed by *cps*14 [1]. The composition of the capsule of *cps2* and *cps*14 is almost identical, the only difference is the absence of rhamnose in *cps*14 [4].

To this date no licensed vaccine against *S. suis* is available in Europe. Autogenous vaccines are widely used in the field but do not provide heterologous protection. Furthermore, protective efficacy of autogenous bacterins varies in association with the infection status of the herd [5]. Different recombinant subunit vaccines have been evaluated in previous experimental studies in pigs including vaccines based on surface antigen one (SAO) [6,7], surface-anchored DNA-nuclease (SsnA) [8], different proteins [9,10] and IgM-degrading enzyme of *S. suis* (Ide_*Ssuis*_) [11,12].

Ide_*Ssuis*_ is a host-specific protease cleaving only porcine IgM [13]. IgM is a strong activator of the classical complement pathway. *S. suis* reduces IgM mediated C3-deposition on the bacterial surface through expression of Ide_*Ssuis*_. *In vitro*, an Ide_*Ssuis*_-deficient mutant is impaired in survival in porcine blood with high levels of IgM binding to the streptococcal surface [14,15]. Active piglet immunization with recombinant Ide_*Ssuis*_ (rIde_*Ssuis*_) is protective against mortality caused by experimental *cps*2 and *cps*9 infection [11,12]. Immunization of piglets with Ide_*Ssuis*_ results in antibodies neutralizing the IgM protease activity.

In this study we investigated immunogenicity of a rIde_*Ssuis*_ vaccine in conventional and CDCD piglets and the protective efficacy against intranasal *cps*14 challenge.

## 2 Materials and methods

### 2.1 Bacterial strains and growth conditions

*S. suis* strain V3117/2 is a *mrp* + *sly* + epf * + sequence type (ST) 1 *cps*14 strain isolated from the brain of a diseased piglet. This strain was used in all *in vivo* and *in vitro* experiments shown in the main figures and is designated as homologous strain in Supplementary data file 3. Strain TW078/11 is a ST1552 *cps*14 strain isolated in the United Kingdom [16]. It was only used in the bactericidal assays with reconstituted blood shown in Supplementary data file 3 and is referred to as heterologous *cps*14.

*Streptococci* were cultured in Todd Hewitt Broth (THB) under microaerophilic conditions (5 % CO_2_) and plated on Columbia agar plates with 6 % sheep blood. Inoculum for intranasal *S. suis* infection was prepared in Tryptic soy broth (TSB). *Escherichia coli* (*E. coli*) strains were cultured in Luria-Bertani (LB) medium with the addition of 100 μg/ml ampicillin. Strains were stored at −80 °C with addition of 20 % glycerine.

### 2.2 Partial sequencing of ide_*Ssuis*_ gene of strain V3117/2

The published primer pair IdeSsuis_con_re and IdeSsuis_con_fo [14] was used to amplify the conserved region of ide_*Ssuis*_ encoding the IgM protease domain (nucleotides 252 to 867 of ide_*Ssuis*_ of *S. suis* strain 10). The amplification product was sequenced using Sanger Cycle Sequencing/Capillary Electrophoresis. Sequence data was submitted to GenBank and is available under accession number PQ497110.

### 2.3 Detection of Ide_*Ssuis*_ in the supernatant of strain V3117/2

Concentrated culture supernatant of *S. suis cps*14 strain V3117/2 was investigated in a Western blot after SDS-PAGE with a polyclonal rabbit serum raised against rIde_*Ssuis*_ of *S. suis cps*2 strain 10 as described previously [13].

### 2.4 Expression and purification of recombinant (r) proteins

Recombinant His-tagged Ide_*Ssuis*_ (rIde_*Ssuis*_) of *cps*2 strain 10 and the truncated His-tagged variant rIde_*Ssuis*__homologue were expressed and purified *via* nickel affinity chromatography as described before [13,14]. The latter was used in the hemolysis assay to cleave IgM *in vitro*.

### 2.5 Virulence-associated gene profiling

A multiplex PCR assay (MP-PCR) was used to detect capsular polysaccharides of *cps*1/14, 2/, 7, 9 and virulence-associated genes in *S. suis* namely muramidase-released protein (*mrp),* extracellular factor *(epf*) and suilysin *(sly)* [17]. As the results of the MP-PCR do not provide differentiation between *cps*1 and *cps*14, amplification and sequencing of the *cps*K gene was conducted essentially as described previously [18].

### 2.6 Vaccination – challenge trial in conventionally raised piglets

Conventionally raised German landrace piglets were obtained from a herd known to be free of *cps*1, *cps*7, *cps*9 and *cps*14 but not *cps*2. This classification is based on intensive screenings for numerous years [9,11].

Females and castrated males were used in the study. Litter mates were distributed equally into two groups (*n* = 9/group; litter-matched design). The piglets were moved into the biosafety facility between 29 and 31 days of age. The unit contained a partially slatted floor with enrichments in the form of toys and ropes. The piglets' lying area was covered with a rubber mat. The light/dark rhythm was maintained by a constant light regime with a light phase of 12 h. The light intensity in the animal area was between 150 and 200 lx at a height of 40 cm above the floor. The floor of the animal area was cleaned daily. Piglets were fed complete feed during monitoring (8 h interval after infection) to record their appetite as part of the scoring system. Fresh drinking water was available *ad libitum*.

Piglets were prime-boost immunized with 0.4 mg rIde_*Ssuis*_ and 20 % Emulsigen as adjuvant (oil-in-water) in the 5th and 7th week of life. Placebo piglets received 1.5 ml PBS containing 20 % Emulsigen. Fourteen days after boost immunization all piglets were challenged intranasally with 1.5 × 10^9^ colony forming units (CFU) of *S. suis cps*14 strain V3117/2. One percent acetic acid (3 ml/piglet) was applied 2 h before intranasal infection for predisposition as described [19].

During the observation period of 14 days following infection, piglets were monitored every 8 h. Body temperature, feed uptake, general condition as well as specific clinical signs of disease such as lameness and opisthotonus were recorded and scored as described [11]. Piglets were classified as morbid if the body temperature reached values ≥40.2 °C. Severe morbidity was defined by body temperature values ≥40.5 °C with additional observation of severe clinical signs of an acute disease such as anorexia or if fever ≥40.2 °C lasted over 24 h.

In case of persisting high fever (≥ 40.5 °C), apathy and anorexia over 24 h as well as in all cases of central nervous system dysfunction or clinical signs of acute polyarthritis combined with recumbency, animals were euthanized for animal welfare reasons. All other piglets were sacrificed 14 days post infection (dpi). Bacteriological, pathological and histopathological investigations were conducted as described [9,11]. Isolates with a *mrp* + epf * *+ sly* *+ cps*1/14*+* genotype were considered isolates of the *cps*14 challenge strain V3117/2. Blood samples were drawn before immunization, 11 days post boost and prior to euthanasia.

### 2.7 Vaccination trial in CDCD piglets

In a second animal experiment, cesarean-derived and colostrum-deprived (CDCD) piglets were raised in a facility with a very high biosafety level and without contact to other piglets. Housing conditions included a levelled floor and autoclaved straw as enrichment but were otherwise similar to the unit used for the experimental infection of the conventional piglets. Female and male piglets were used and the proportion of sexes per group was close to equal. CDCD piglets were prime-boost immunized with a vaccine generated by our industrial partner (Ceva Innovations GmbH). It included a confidential rIde_*Ssuis*_ variant and an oil-in-water adjuvant. Control CDCD piglets received only the adjuvant. Vaccination of CDCD piglets was conducted through intramuscular injection in the neck at an age of 4 and 6 weeks of life (*n* = 8/group). Blood samples were taken 14 days after boost immunization.

### 2.8 Bactericidal assays

Bactericidal assays were conducted as described [13]. Briefly, 500 μL of freshly drawn heparinized blood (16 I. U. heparin/mL) were infected with 3 × 10^6^ CFU of *S. suis* and incubated on a rotator for 120 min at 37 °C. To determine the specific bacterial load, serial dilutions were plated on blood agar plates at *t* = 0 min and *t* = 120 min. Survival factors were calculated dividing CFU at t = 120 by CFU at *t* = 0.

In bactericidal assays with whole blood of CDCD piglets, the *S. suis* inoculation dose was reduced to 1.5 × 10^4^ CFU. Preliminary results showed that reduction of the number of *streptococci* in experiment with blood of CDCD piglets is necessary to prevent bacterial overgrowth.

### 2.9 Bactericidal assays with reconstituted blood

Bactericidal assays with reconstituted blood were conducted as described before except that serum and not plasma was used to reconstitute blood [16]. Briefly, blood cells for reconstitution were harvested from freshly drawn heparinized (16 I. U. heparin/mL) blood samples from piglets. For reconstitution of blood, 100 μL serum and 100 μL blood cells were mixed. Reconstituted blood samples were inoculated with 2.4 × 10^5^ CFU of *S. suis* strains V3117/2 or TW078/11 and incubated rotating at 37 °C for 2 h. The specific bacterial content and the survival factor were determined as described under bactericidal assays.

### 2.10 Detection of α-rIde_*Ssuis*_ IgG and α-*cps*14 IgM

Levels of serum IgM binding to immobilized, formaldehyde-inactivated *S. suis cps*14 (strain V3117/2) was determined in a whole cell ELISA as published before [16]. A peroxidase-conjugated, goat anti pig IgM antibody (PA1–84625, Thermo Scientific, Schwerte, Germany) is used to detect IgM binding to the surface of inactivated *streptococci* in this ELISA. Detection of IgG antibodies binding to immobilized rIde_*Ssuis*_ was conducted as described [11,12]. Serum of a piglet immunized with rIde_*Ssuis*_ in a previous study was defined to contain 100 ELISA units and served as reference serum as in our previous studies.

### 2.11 Hemolysis assay

In a previous study [15], porcine antisera raised against ovine erythrocytes were used to induce complement-dependent hemolysis. Addition of rIde_*Ssuis*__homologue was shown to prevent this hemolysis [15]. In this study, we expanded this assay to quantitatively access neutralization of the IgM protease activity by porcine sera.

Specifically, sheep blood was washed three times with PBS and diluted up to a 2 % ovine erythrocyte suspension. Each well of a V-bottom 96 well plate was filled with 100 μL of the 2 % sheep erythrocyte suspension. Addition of water leading to complete lysis of the erythrocytes defined 100 % hemolysis, addition of physical saline solution defined 0 % hemolysis. Addition of porcine serum drawn 7 days after vaccination with ovine erythrocytes (αEry) served as positive control for hemolysis. This serum was also incubated with rIde_*Ssuis*__homologue for 1.5 h at 37 °C to allow cleavage of IgM and was subsequently used as negative control.

Sera of rIde_*Ssuis*_-prime-boost-vaccinated or placebo-treated piglets drawn 11 days after boost vaccination were diluted 1:10 and incubated rotating with 2 μg rIde_*Ssuis*__homologue/100 μL serum dilution [13] for 1 h at 4 °C for potential neutralization of the IgM protease. Porcine serum drawn 7 days after vaccination with ovine erythrocytes (αEry) was added, incubated rotating for 1.5 h at 37 °C to allow possible cleavage of IgM-α-ovine erythrocytes through rIde_*Ssuis*__homologue and subsequently transferred to the V-bottom 96 well plate filled with 2 % sheep erythrocyte suspension.

After incubation for 20 min at 37 °C with gentle shaking for possible lysis of the erythrocytes, the 96 well plate was centrifuged for 5 min at 1000*g*. Supernatants were transferred to a flat bottom 96 well plate and optical density was measured at 425 nm. The experiment was repeated three times.

### 2.12 Statistical analysis

Differences between two groups were analyzed with the Mann-Whitney *U* test or in case of normal distribution with the unpaired *t*-test. Comparison of two time point values within the same group was carried out using the Wilcoxon test. In case of three time point values the Friedman test and subsequently the Dunn's multiple comparisons test were conducted. Data presented in the Kaplan-Meier-diagrams were analyzed with the Gehan-Breslow-Wilcoxon test. Means and standard deviations of the results are shown. All statistical tests were conducted with GraphPad Prism 7.01 software. Probabilities lower than 0.05 were considered significant (*p < 0.05, **p < 0.01, ***p < 0.001).

## 3 Results

### 3.1 Prime-boost-immunization with rIde_*Ssuis*_ induces specific α-rIde_*Ssuis*_ antibodies in piglets

**Fig. 1:**
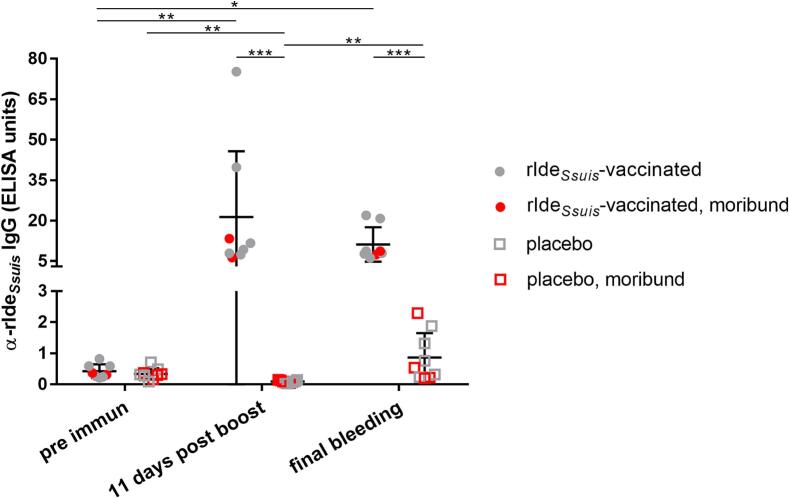
rIde_*Ssuis*_ vaccination induces specific serum IgG antibodies in prime-boost-vaccinated piglets. Levels of IgG antibodies binding to immobilized rIde_*Ssuis*_ were determined in an ELISA. Sera of rIde_*Ssuis*_-vaccinated (*n* = 8) and placebo-treated piglets (*n* = 9) were collected pre immunization, 11 days post boost and prior euthanasia after challenge at the indicated time points post infection. Data of one rIde_*Ssuis*_-vaccinated piglet were excluded from the graph and statistical analysis as the final bleeding serum of this animal was not available (see Table S1). Samples of piglets which succumbed to *S. suis* infection are marked in red. Serum of a previously rIde_*Ssuis*_-prime-boost vaccinated piglet was used as standard to define 100 ELISA units [12]. Mean values are indicated by horizontal lines, standard deviations by error bars. Statistical analyses were conducted with the Mann-Whitney *U* test (placebo *vs.* rIde_*Ssuis*_-vaccinated) and the Friedman test with a subsequent Dunn's multiple comparisons test (comparison of time points within one group). Significant differences are indicated (* *p* < 0.05, ** *p* < 0.01, *** *p* < 0.001). (For interpretation of the references to colour in this figure legend, the reader is referred to the web version of this article.)

Previous vaccination studies demonstrated highly significant elevated IgG levels against Ide_*Ssuis*_ after active immunization of piglets with the purified recombinant antigen with mean ELISA units above 60 [11,12]. In this study conventionally raised piglets of the same herd were prime and boost vaccinated with rIde_*Ssuis*_ at the age of 4.5 and 6.5 weeks of life, respectively. The original herd is not free of *S. suis*, but free of *cps*14. IgG antibodies binding to rIde_*Ssuis*_ were determined in the same ELISA as in the previous studies [11,12]. α- rIde_S*suis*_ IgG rose from a mean of 0.4 ELISA units (S.D. = 0.2) before immunization to a mean of 19.6 ELISA units (S.D. = 23.4) 11 days post boost. In the respective placebo-treated group IgG-α-rIde_*Ssuis*_ levels remained low with a mean of 0.3 (S.D. = 0.2) and 0.1 (S.D. = 0.0) ELISA units pre immunization and post boost, respectively (Fig. 1, Table S1).

### 3.2 Survival of *S. suis cps*14 in blood of rIde_*Ssuis*_-vaccinated piglets

Previous studies demonstrated reduction of survival of *S. suis cps*2 and *cps*9 in blood of rIde_*Ssuis*_ vaccinated piglets [11,12]. In this study, we focused on protection against a *cps*14 strain belonging to clonal complex 1, which also harbors the *cps*2 strain of the former rIde_*Ssuis*_ vaccination study [12,20]. The region of the ide_*Ssuis*_ gene of the *cps*14 strain V3117/2 encoding the IgM protease domain was sequenced and found to have a high degree of identity to the published sequence of *S. suis cps*2 strains 10 and P1/7 (98 % identity, nucleotides 252 to 867). Furthermore, Ide_*Ssuis*_ was detected in the concentrated supernatant of strain V3117/2 using a polyclonal antiserum raised against rIde_*Ssuis*_ of *S. suis* strain 10 (results not shown).

**Fig. 2:**
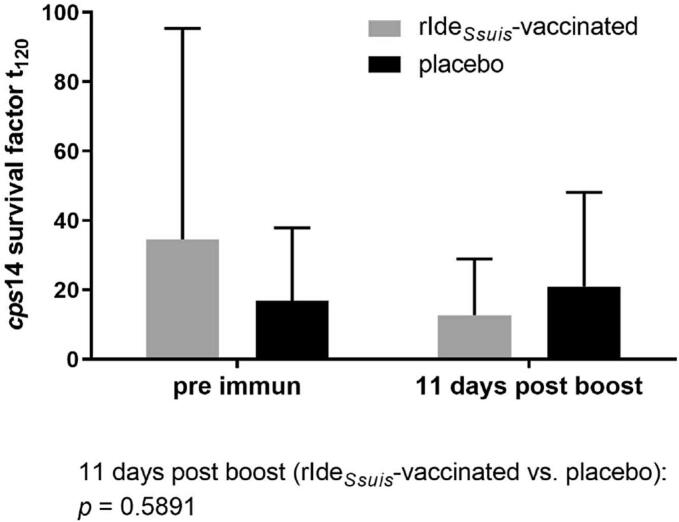
Survival of *S. suis cps*14 strain V3117/2 in blood of rIde_*Ssuis*_-prime-boost-vaccinated and respective placebo-treated litter mates. Piglets were either rIde_*Ssuis*_-vaccinated or placebo-treated (n = 9/group) and survival of *S. suis cps*14 strain V3117/2 was investigated in their blood *ex vivo* prior to immunization and 11 days post boost. Bars and error bars represent mean values and standard deviations, respectively. The survival factor represents the ratio of CFU at 120 min to CFU at time point zero. No significant differences were obtained using the Mann-Whitney-*U* test (rIde_*Ssuis*_-vaccinated *vs.* placebo) and the Wilcoxon test (comparison of time points within the group). The *p* value obtained comparing of both groups at the time point 11 days post boost is shown below the diagram.

We investigated survival of *S. suis cps*14 strain V3117/2 in a bactericidal assay including blood of conventionally raised piglets prior to and post rIde_*Ssuis*_-prime-boost-immunization. The mean bacterial survival factor decreased from a mean of 34.6 (S.D. = 60.8) pre immunization to a mean of 12.7 (S.D. = 16.3) post boost in blood of rIde_*Ssuis*_-vaccinated piglets. In comparison to the immunized piglets, the mean survival factor in blood samples of placebo-treated litter mates was substantially higher with 20.9 (S.D. = 27.2) (Fig. 2, Table S1) at the latter time point. These results suggest a reduction of proliferation of the *cps*14 strain in porcine blood through immunization with rIde_*Ssuis*_*.*

### 3.3 Results of the *S. suis cps*14 challenge experiment suggest delayed onset of disease and prevention of bacterial dissemination

**Fig. 3:**
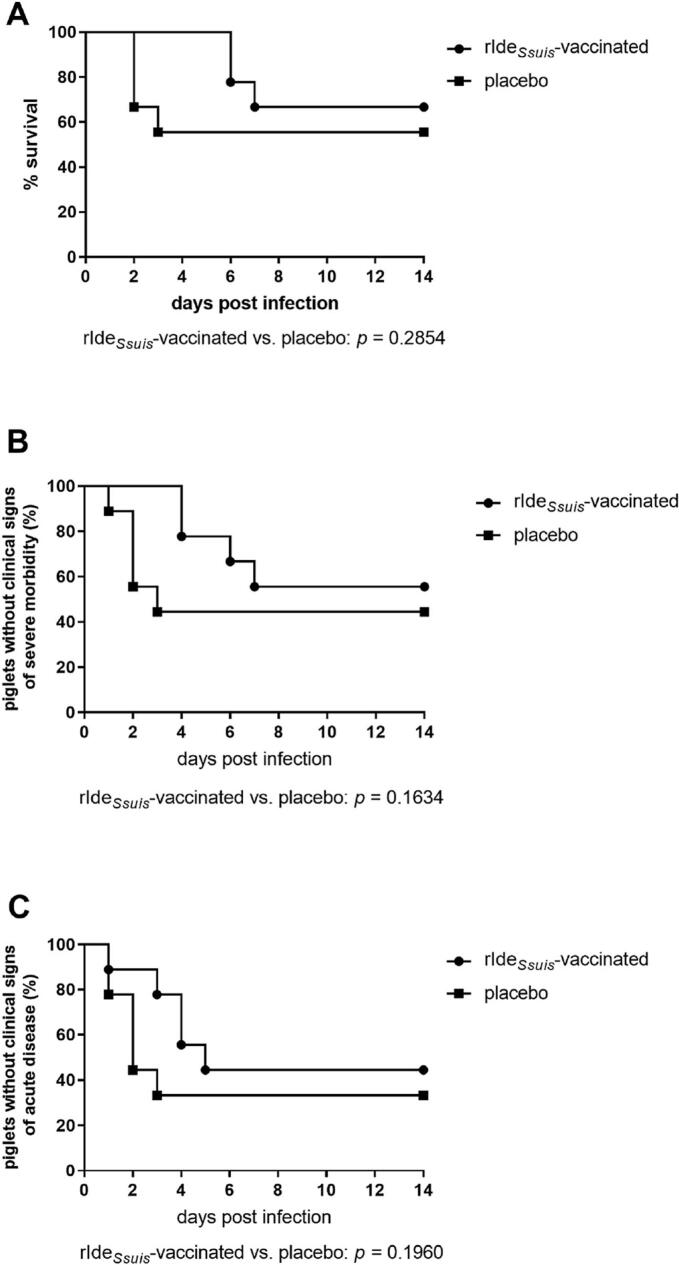
Mortality (A), severe morbidity (B) and morbidity (C) after infection with *S. suis cps*14 strain V3117/2 in rIde_*Ssuis*_-vaccinated and placebo-treated piglets. Eighteen piglets were challenged intranasally with 1.5 × 10^9^ CFU of *S. suis cps*14 strain V3117/2 14 days after rIde_*Ssuis*_ boost immunization. **A** In case of high fever (≥ 40.5 °C), apathy and anorexia persisting over 24 h as well as in all cases of central nervous system dysfunction or clinical signs of acute polyarthritis/recumbency, animals were euthanized for animal welfare reasons. All surviving piglets were sacrificed 14 days post infection. **B** Severe morbidity was defined as a body temperature ≥ 40.2 °C lasted over 24 h or if the body temperature reached ≥40.5 °C combined with reduced feed intake. **C** Piglets were classified as morbid as soon as the body temperature ≥ 40.2 °C or/and severe clinical signs of an acute disease were observed. The *p* values analyzed with the Gehan-Breslow-Wilcoxon test are shown below the Kaplan-Meier diagrams.

Two weeks after the prime-boost vaccination with rIde_*Ssuis*_ piglets were challenged intranasally with *S. suis cps*14 strain V3117/2. Severe clinical signs of disease were observed in five of nine placebo-treated piglets within the first three days post infection. Clinical signs included high fever (*n* = 2), lameness (*n* = 1), no feed intake (n = 1) and faintness (*n* = 3) (Fig. 3B). Four piglets of this group developed an acute polyarthritis and were euthanized for animal welfare reasons (three piglets on day 2 and one piglet on day 3 after infection) (Fig. 3A and Table 1). In contrast, only mild clinical signs were observed in piglets of the rIde_*Ssuis*_-vaccinated group during the first 3 days (Fig. 3B).

**Table 1:** Assessment of morbidity, mortality and clinical signs after intranasal *S. suis cps*14 challenge.

Immuni- zation antigen	Morbidity	Mortality	Mean clinical score^a^ (SD)	Clinical signs			Max. body temperature		
				CNS^b^	Lameness	No feed intake	<40	40–40.2	>40.2
rIde_*Ssuis*_	5/9	3/9	10.8 (11.0)	1/9	2/9	5/9	4/9	1/9	4/9
Placebo	6/9	4/9	13.1 (11.6)	0/9	5/9	4/9	3/9	0/9	6/9

a For the detailed clinical scoring system see Table S1 by Rieckmann et al.*,* [11]

b Signs of central nervous system (CNS) dysfunction such as convulsions and opisthotonus.

On day 4 post infection four of nine rIde_*Ssuis*_-vaccinated piglets showed also severe morbidity namely fever (*n* = 4), reduced feed intake (*n* = 2) or lameness (*n* = 1) (Fig. 3B). Six days post infection two piglets of this group were euthanized, one due to acute signs of polyarthritis and one due to a persisting disturbed general condition. The following day one further piglet of the immunized group was euthanized for reasons of central nervous system dysfunction (Fig. 3A and Table 1).

In line with the clinical signs of lameness, the *S. suis cps*14 challenge strain V3117/2 was recovered from two or more joints of five piglets of the placebo-treated group. Four of these piglets were euthanized before the end of the observation period. The fifth piglet did not reach humane end points throughout this trial although the *S. suis cps*14 challenge strain V3117/2 was reisolated from two of four investigated joints and the mitral valve of this piglet (Table 2). The challenge strain was recovered from several joints and inner organs in two placebo-treated piglets indicating substantial dissemination (Table 2).

**Table 2:** Reisolation of the challenge strain from piglets after intranasal challenge with *S. suis cps*14 strain V3117/2.

Immuni-zation antigen	Number of piglets positive for the isolation of the challenge strain in an inner organ^b^ or in serosa^d^ or in joint fluid^f^	Number of piglets positive for the isolation of the challenge strain in two or more inner organs^b^ or in serosae^d^ or in joint fluids^f^ or a combination of these sites	Number of piglets in which the *S. suis cps*14 challenge strain^a^ was isolated from							
			Tonsils	Lung^c^	Serosa^d^	Spleen	Liver	Brain/ CSF^e^	Joint fluid^f^	Endocard
rIde_*Ssuis*_	3/9	1/9	6/9	0/9	0/9	0/9	0/9	2/9	1/9	0/9
Placebo	5/9	5/9	3/9	3/9	1/9	1/9	2/9	1/9	5/9	2/9

a The challenge strain was identified by PCR.

b Inner organ refers to lung, spleen, liver, brain/CSF or endocard but not the tonsils.

c One cranial lobe was investigated.

d Pleural, peritoneal or pericardial cavity.

e Cerebrospinal fluid.

f Punctures of both tarsal and carpal joints were investigated in each animal. In case of lameness additional joint punctures of the respective limb were screened.

In the immunized group the challenge strain was recovered from two joints of the piglet suffering from polyarthritis, and from the brain/cerebrospinal fluid (CSF) of the piglet which showed signs of central nervous system disorder. The third piglet was euthanized due to persisting disturbed general condition. Tremor and recumbency was observed prior to euthanasia but no severe clinical signs of central nervous disorder. Bacteriological investigations of this piglet revealed that *streptococci* had reached the brain/CSF (Table 2). Noteworthy, bacterial dissemination defined as detection of the challenge strain in at least two inner organs, was recorded in one vaccinated piglet only whereas this was the case for five placebo-treated piglets.

Histopathological scoring did not reveal a difference between both groups with group means of 2.8 and 2.9 for the immunized and the placebo group, respectively (Table 3).

**Table 3:** Scoring of fibrinosuppurative lesions of piglets challenged with *S. suis cps*14 strain V3117/2.

Immuni-zation antigen	Piglets without lesions^a^	Piglets with lesions in two or more locations^a^	Brain			Serosae				Joint				Spleen and liver				Lung				Heart				ω^f^
			Meningitis, chorioiditis			Pleuritis or peritonitis or pericarditis				Synovialitis				Splenitis^b^ or hepatitis				Pneumonia				Endocarditis				
			5^c^	3^d^	1^e^	4^c^	2^d^	1^e^		4^c^	2^d^	1^e^		4^c^	2^d^	1^e^		4^c^	2^d^	1^e^		4^c^	2^d^	1^e^		
rIde_*Ssuis*_	0/9	8/9	0/9	1/9	4/9 g	0/9	3/9	4/9		2/9	2/9	0/9		0/9	3/9	4/9		1/9	7/9	0/9		0/9	2/9	3/9		**2.8**
Placebo	1/9	6/9	0/9	1/9	2/9 h	0/9	1/9	3/9		2/9	4/9	0/9		0/9	5/9	3/9		1/9	5/9	0/9		1/9	1/9	0/9		**2.9**

a Only fibrinosuppurative lesions are considered. Individual single perivascular neutrophils are not counted.

b Neutrophilic accumulation of the splenic red pulp.

c Scoring of 4 and 5 indicates moderate to severe diffuse or multifocal fibrinosuppurative inflammations.

d Scoring of 2 and 3 indicates mild focal fibrinosuppurative inflammation.

e Individual single perivascular neutrophils received a score of 1.

f ω = Σscore_max_/n_animals_.

g Plexus choroiditis.

h Plexus choroiditis in one of two piglets.

Immunization with rIde_*Ssuis*_ did not lead to complete protection against morbidity and mortality caused by an intranasal challenge with *S. suis cps*14 strain V3117/2. However, immunization suggests partial protection as the onset of severe disease and mortality following *S. suis* infection was delayed in vaccinated piglets compared to the placebo-treated group.

### 3.4 Determination of α-Ide_*Ssuis*_ antibodies neutralizing the IgM protease activity

To determine whether vaccination with rIde_*Ssuis*_ and resulting α-rIde_*Ssuis*_ antibodies led to neutralization of IgM-proteolysis, post boost serum samples of nine rIde_*Ssuis*_-prime-boost-vaccinated piglets and the respective placebo-treated group were investigated in a hemolysis assay measuring neutralization of IgM-proteolysis through rIde_*Ssuis*__homologue. The experiment was conducted three times and means include values of all experiments, which are shown in Table S2. Application of sera collected 10 days after rIde_*Ssuis*_-boost-vaccination resulted in a group mean of 52.6 % hemolysis (S.D. = 36.8) indicating induction of rIde_*Ssuis*_ neutralizing antibodies. In comparison, sera of placebo-treated piglets showed significantly lower values with mean of 8.1 % hemolysis (S.D. = 2.1).

**Fig. 4:**
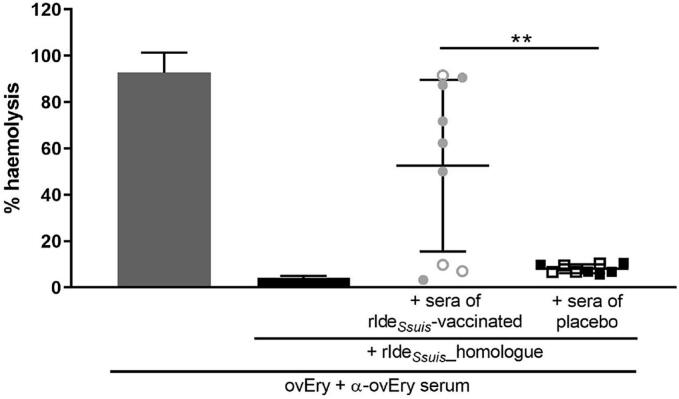
Neutralization of the IgM protease activity of rIde_*Ssuis*__homologue through sera of rIde_*Ssuis*_-vaccinated piglets as determined in a hemolysis assay with porcine anti-erythrocyte serum. Sera drawn from rIde_*Ssuis*_*-* vaccinated and placebo-treated piglets (n = 9/group) were preincubated with rIde_*Ssuis*__homologue to allow binding of anti-rIde_*Ssuis*_ antibodies. Next, porcine serum drawn 7 days after vaccination with ovine erythrocytes (α-ovEry serum) was added to induce cleavage of IgM-α-ovEry in the absence of antibodies neutralizing rIde_*Ssuis*__homologue. Components were added to washed sheep erythrocytes (ovEry) and hemolysis was determined subsequently. Hemolysis assay was conducted three times in independent experiments. Results (primary data) of these experiments are listed in Table S2. Empty circles and squares stand for early euthanized piglets due to reaching of humane end points, filled circles and squares present survivors. The value for each point is calculated as the mean % of hemolysis in the three experiments of the respective serum. Group means are calculated as mean of all values of the three experiments of the respective group. Bars and error bars represent mean values and standard deviations, respectively. Significant differences were determined using unpaired *t*-test (rIde_*Ssuis*_-vaccinated *vs.* placebo). Significances are indicated (** *p* < 0.01).

Six of nine sera of rIde_*Ssuis*_-vaccinated piglets showed a strong neutralization of the IgM protease activity indicated by hemolysis values above 45 %, whereas hemolysis values of three sera remained below 12 % (Fig. 4).

Two of these three piglets were euthanized due to severe clinical signs of disease before the end of the observation period. One rIde_*Ssuis*_-vaccinated piglet developed an acute polyarthritis despite high neutralization capability in the hemolysis assay and was euthanized prematurely.

### 3.5 rIde_*Ssuis*_ immunization of CDCD piglets results in killing of *S. suis cps*14 in blood *in vitro*

**Fig. 5:**
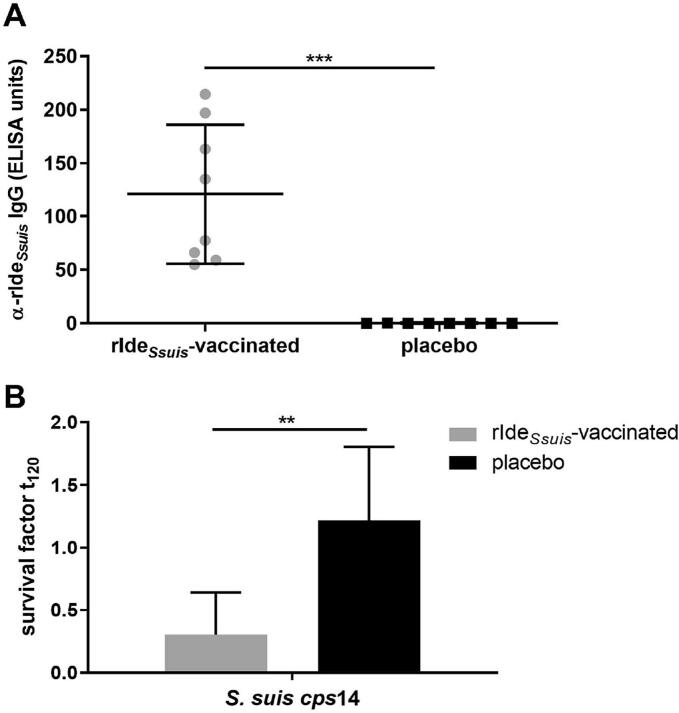
rIde_*Ssuis*_ prime-boost-vaccination induces high specific serum IgG antibodies and bactericidal immunity against *S. suis cps*14 strain V3117/2 in blood of vaccinated CDCD piglets. **A** IgG binding to rIde_*Ssuis*_ was determined in serum of 8-week-old rIde_*Ssuis*_-vaccinated and placebo-treated CDCD piglets (n = 8/group) 14 days after boost immunization in an ELISA. **B** Whole blood drawn at the same time point was inoculated with 1.5 × 10^4^ CFU of *S. suis cps*14 strain V3117/2 (n = 8/group) and incubated rotating for 120 min. The survival factor represents the ratio of CFU at 120 min to CFU at time point zero. **A** **+** **B**: Mean values are indicated by horizontal lines, standard deviations by error bars. Statistical analyses were conducted with the unpaired t-test. Significant differences are indicated (** *p* < 0.01, *** *p* < 0.001).

In another vaccination trial a similar immunization regime was applied to CDCD piglets which were raised in a high biosecurity unit to ensure that they remain free of *S. suis* and other porcine pathogens. The vaccine included a modified rIde_*Ssuis*_ variant and a different water-in-oil adjuvant. At the age of 8 weeks, 14 days after boost immunization, α-rIde_*Ssuis*_ IgG were determined and revealed significantly higher antibody levels in serum samples of rIde_*Ssuis*_-vaccinated CDCD piglets with a mean of 120.2 ELISA units (S.D. = 60.2) compared to the placebo-treated CDCD piglets with a mean of 0.2 ELISA units (S.D. = 0.1) (Fig. 5).

Blood samples drawn 14 days after rIde_*Ssuis*_-boost vaccination or placebo-treatment in CDCD piglets were infected with *S. suis cps*14 *in vitro*. A significantly lower survival factor of *S. suis cps*14 strain V3117/2 was recorded in blood samples of the immunized CDCD piglets with a mean value of 0.30 (S.D. = 0.34) compared to placebo-treated CDCD piglets with a mean value of 1.22 (S.D. = 0.59) (Fig. 5).

## 4 Discussion

*S. suis cps*14 is the second main cause for human *S. suis* infections worldwide and plays a major role in *S. suis* disease in pigs in the United Kingdom and North America [1]. *S. suis cps*14 strains isolated from pigs in North America within diagnostic services were all classified as pathogenic and belonged to ST1 [21]. Association of *cps*14 with disease was also recorded in an older European study including *cps*14 isolates from the UK [3].

Similar to our previous experimental *S. suis cps*14 study [9] we observed severe morbidity in approximately half of the non-vaccinated piglets after intranasal *cps*14 challenge. Induction of severe disease in conventional piglets after intranasal application underscores the comparable high virulence of this *cps*14 strain. Noteworthy, piglets of the same herd did not show clinical signs of disease after intranasal application of *cps*9 strain [22]. Because of the high virulence and zoonotic potential, it is important to include *S. suis cps*14 in vaccination studies.

Here, we investigated a rIde_*Ssuis*_ vaccine and evaluated it's protective efficacy in an intranasal *cps*14 challenge. Ide_*Ssuis*_ was shown to be a highly protective antigen. Seele *et al.* [12] showed that rIde_*Ssuis*_-prime-boost-immunization after weaning leads to protection against mortality and morbidity in *cps*2 challenged piglets. Furthermore, rIde_*Ssuis*_*-*prime-boost-boost immunization resulted in protection against mortality induced through challenge with a highly virulent *cps*9 strain [11]. Levels of α-rIde_*Ssuis*_ antibodies were determined in all rIde_*Ssuis*_ vaccination studies with the same protocol and the same standard serum. Piglets in this study developed mean α-rIde_*Ssuis*_ levels of only 19.6 ELISA units (S.D. = 23.4) whereas mean α-rIde_*Ssuis*_ levels obtained 70.4 (S.D. = 35.5) and 64.4 (S.D. = 43.9) ELISA units in the rIde_*Ssuis*_-vaccinated groups of the studies by Seele *et al.* [12] and Rieckmann *et al.* [11], respectively. The reason for the lower level of α-rIde_*Ssuis*_ IgGs in this study is not known but might be related to health problems in association with diarrhoea in these piglets during the acclimatization to the experimental facility after weaning (data not shown). In any case, we speculate that the lack of significance of protection might be related to the lower Ide_*Ssuis*_ specific IgG level. Due to the high degree of identity of the IgM protease domain of the ide_*Ssuis*_ variants of *S. suis* strains V3117/2 and 10, it appears unlikely that differences in the amino acid sequence are responsible for the low protection observed in this study. However, we cannot rule out that Ide_*Ssuis*_ shows differences in expression, localisation and accessibility to antibodies between the two strains that are relevant for the protective efficacy of the vaccine.

We measured levels of α-rIde_*Ssuis*_ IgG in sera drawn from moribund and surviving piglets immediately prior to euthanasia. Similar to our previous *cps*9 study [11], the data in Fig. 1 indicates that the experimental *cps*14 infection does not booster α-rIde_*Ssuis*_ IgG levels in vaccinated piglets. Though a slight decline of α-rIde_*Ssuis*_ IgG was observed in single piglets, none of the final samples of the vaccinated piglets showed levels comparable to the levels of the control piglets with values below 5 ELISA units. Furthermore, there does not appear to be a difference between moribund piglets and survivors in final α-rIde_*Ssuis*_ IgG levels. Thus, we consider it unlikely that the delayed onset of disease in vaccinated piglets is related to a depletion of α-rIde_*Ssuis*_ IgG.

Immunization of CDCD piglets resulted in high levels of IgG-α-rIde_*Ssuis*_ with a mean of 120.2 ELISA units (S.D. = 60.2). Utilisation of a different adjuvant might have influenced the antibody response. However, as previous rIde_*Ssuis*_ vaccination studies [11,12] with higher specific IgG levels included also vaccines with 20 % Emulsigen, the use of Emulsigen is for itself not a reasonable explanation for lower level of recorded α-rIde_*Ssuis*_ IgG levels in piglets of the *cps*14 challenge trial (Emulsigen was only used in the vaccine in conventional piglets). In any case, higher α-rIde_*Ssuis*_ IgG levels in vaccinated CDCD piglets were associated with immunity leading to killing of *S. suis cps*14 in blood of vaccinated piglets. Studies on *S. suis* carried out with conventionally raised piglets may be influenced by immunity elicited by natural infection and maternal Ig. Experimental studies with CDCD piglets exclude such effects [23]. This study demonstrates for the first time that immunity elicited by other colonizing *S. suis* strains (or maternal immunity) is not crucial for induction of bactericidal immunity by rIde_*Ssuis*_ vaccination as determined in the bactericidal assay. This is very important as it is reasonable to speculate that there is a synergistic effect of IgM elicited by natural infection and vaccination induced IgG antibodies neutralizing Ide_*Ssuis*_ activity on the bacterial surface. However, the comparison of the experiments with the conventional and CDCD piglets is limited by the fact that not only the status of the piglets was different but also the vaccine composition and the housing conditions. Furthermore, CDCD piglets were not challenged experimentally with *S. suis* cps14. It remains to be shown if CDCD piglets are protected against disease induced by experimental *S. suis cps*14 infection, though the data of the bactericidal assay in Fig. 5 suggest protection against *S. suis cps*14 bacteremia. Furthermore, a future study evaluating different adjuvants for an Ide_*Ssuis*_ vaccine is reasonable, as a recent *S. suis* study indicated that Emulsigen is not the best adjuvant, at least not for a bacterin [24].

Increase of serum IgM binding to the surface of *S. suis cps*14 within 14 days post infection and killing of the homologous and a heterologous *cps*14 strain after intranasal *cps*14 challenge in reconstituted blood (Fig. S1 in Supplementary data file 3) suggests induction of IgM by experimental mucosal infection and is in agreement with IgM cross-reacting between closely related *S. suis* strains [16].

We hypothesize that induction of rather low α-rIde_*Ssuis*_ IgG levels in this study is related to the lack of significant protection against morbidity and mortality after *cps*14 challenge. However, α-rIde_*Ssuis*_ IgG levels do not seem to correlate with clinical scores recorded after experimental infection. Furthermore, a putative threshold for α-rIde_*Ssuis*_ IgG levels required to ensure protection remains to be elusive [11].

Functionality of antibodies directed against *S. suis cps*14 and against rIde_*Ssuis*_ were investigated in a bactericidal assay and a hemolysis assay, respectively. Antibody- and complement dependent opsonophagocytosis play an important role for bactericidal immunity [25]. As killing of *S. suis cps*14 was observed in association with high levels in CDCD piglets, we propose direct induction of opsonophagocytosis by α-rIde_*Ssuis*_ IgG antibodies. However, neutralization of the IgM protease activity as shown for six out of nine individuals of the immunized group, might also play a role for mediating protection.

Two of three vaccinated piglets with insufficient neutralization also showed proliferation of *streptococci* in blood *in vitro*. These two piglets were euthanized early after the challenge due to severe *S. suis* disease which is in accordance with an important role of IgM neutralization in protection.

Piglets of the immunized group which showed neutralization of rIde_*Ssuis*_ in the hemolysis assay and killing of planktonic *streptococc*i in the bactericidal assay passed the *cps*14 infection with no or mild unspecific clinical signs of disease (*n* = 3).

The remaining three individuals of the immunized group which neutralized rIde_*Ssuis*__homologue in the hemolysis assay but failed to kill planktonic *streptococc*i in the bactericidal assay, showed various degrees of clinical signs after infection with *S. suis cps*14. It is reasonable to speculate that opsonophagocytosis was not sufficient to control bacteraemia in these single piglets.

*S. suis* disease leads to suffering and substantial economic losses in the swine industry. A (cross)protective vaccine to combat *S. suis* disease is strongly desired. In a previous study [9], we evaluated a vaccine composed of six immunogens and a following *cps*14 challenge with sobering results underlining the difficulty of generating a well-designed vaccination protocol with result of effective protection against *S. suis* in general and against *cps*14 in particular.

## 5 Conclusions

Prime-boost immunization of conventional weaning piglets with impaired health with a vaccine including rIde_*Ssuis*_ and an oil-in-water adjuvant might not result in significant protection against *S. suis cps*14 in association with comparable low levels of α-rIde_*Ssuis*_ IgG. Vaccination with rIde_*Ssuis*_ elicits antibodies neutralizing the IgM protease activity and leading to a significant increase of IgM-mediated complement activation as determined in a hemolysis assay with porcine anti-erythrocyte serum. Prime-boost-vaccination of healthy CDCD piglets with a rIde_*Ssuis*_ vaccine containing an oil-in-water adjuvant induces high specific serum IgG antibody levels and bactericidal immunity against *S. suis cps*14 indicating that adaptive or maternal immunity against other *S. suis* antigens is not crucial for bactericidal immunity induced by rIde_*Ssuis*_ vaccination.

## Ethical statement

Piglets in the challenge experiment were infected experimentally and cared for in accordance with the principles outlined in the EU Directive 2010/63/EU. All animal experiments or samplings were in accordance with the principles outlined in the European Convention for the Protection of Vertebrate Animals Used for Experimental and other Scientific Purposes and the German Animal Protection Law (Tierschutzgesetz). The challenge experiment of this study was approved by the Saxony Regional Office (permit no. TVV37/17).

The vaccination trial in CDCD piglets was approved by the Central Authority for Scientific Procedures on Animals according to the Dutch law on animal experimentation in accordance with current legislation and fostering the principles of the 3 Rs of animal testing: replacement, reduction, and refinement.

The animal experiment was also approved by the Ethical Committee of Wageningen Bioveterinary Research (The Netherlands) under permit number 2016054 and the Dutch Commission for Animal Studies under the project number AVD401002015140.

## CRediT authorship contribution statement

**L. Mayer:** Writing – original draft, Visualization, Validation, Methodology, Investigation, Formal analysis, Data curation. **C. Liedel:** Writing – review & editing, Methodology, Investigation. **K. Klose:** Writing – review & editing, Methodology, Investigation. **A. de Greef:** Writing - review & editing, Methodology, Investigation. **K. Rieckmann:** Writing – review & editing, Supervision, Methodology. **C.G. Baums:** Writing – review & editing, Supervision, Project administration, Methodology, Investigation, Funding acquisition, Data curation, Conceptualization.

## Declaration of competing interest

Research of CGB was funded by CEVA Innovations GmbH. CGB is the inventor of a patent for a vaccine composition including Ide_*Ssuis*_ (*e.g.* US Patent # 12,036,275). The assignee of this patent is Ceva Santé Animale S.A. (Libourne).

## Data Availability

Data will be made available on request.

## Appendix A Supplementary data

Table S1: Individual clinical scores after cps14 challenge and primary data of bactericidal assays and α-rIdeSsuis IgG Image 1

Table S2: Primary data of hemolysis assays Image 2

Fig. S1 Image 3
