# Supplementary material for: Immunogenicities of vaccines including the immunoglobulin M-degrading enzyme of Streptococcus suis, rIdeSsuis, and protective efficacy against serotype 14 in piglets
Source: Vaccine X. 2024 Nov 21;21:100590. doi: 10.1016/j.jvacx.2024.100590 (PMC11629322; doi:10.1016/j.jvacx.2024.100590)
Supplement: Supplementary file 1 — Table S1: Individual clinical scores after cps14 challenge and primary data of bactericidal assays and α-rIdeSsuis IgG [file mmc1.pdf]

Supplementary data

Table S1: Individual clinical scores after *cps*14 challenge and primary data of bactericidal assays and α-rIde$_{Ssuis}$ IgG.

Immuni-zation antigen	piglet number	α-rIde$_{Ssuis}$ IgG (ELISA units)				Bactericidal Assay (survival factor)		highest clinical score[a] reached after challenge (dpi[b])
		pre immuni-zation	11 days post boost	morbi-bund piglets (2 to 7 dpi[b])	survi-ving piglets (14 dpi[b])	pre immuni-zation	11 days post boost	
rIde$_{Ssuis}$	4545	0.31	13.35	7.41		11.62	9.28	**25** (7)
	5546	0.21	9.28		7.97	1.91	49.30	**3** (12)
	5603	0.26	11.66		7.68	64.71	0.02	**8** (12)
	5648	0.24	7.40		6.14	30.24	0.00	**1** (11)
	5662	0.37	6.32	8.70		1.28	15.21	**25** (6)
	5733	0.59	7.93		8.71	1.99	0.00	**1** (1)
	5799	0.24	5.00	- [c]		6.69	18.71	**25** (6)
	5830	0.82	39.83		20.83	8.52	21.78	**8** (4)
	5875	0.59	75.27		21.96	198.57	0.15	**1** (0)
placebo	4546	0.16	0.04	0.00		63.68	60.25	**25** (2)
	5547	0.49	0.10		0.52	3.10	2.86	**25** (2)
	5600	0.33	0.13	0.00		43.13	39.09	**25** (2)
	5649	0.32	0.14		0.00	2.63	0.00	**0** (-)
	5663	0.71	0.05		0.66	2.95	0.00	**2** (3)
	5732	0.37	0.15	0.00		6.34	2.77	**25** (3)
	5798	0.28	0.03		0.00	4.33	71.55	**3** (4)
	5831	0.30	0.09	0.05		16.21	26.45	**25** (2)
	5877	0.09	0.07		0.56	2.04	5.19	**10** (3)

[a] For the detailed clinical scoring system see Table S1 by Rieckmann *et al.*.2019

[b] days post infection

[c] no serum available

Rieckmann K, Seydel A, Klose K, Alber G, Baums CG, Schütze N. Vaccination with the immunoglobulin M-degrading enzyme of *Streptococcus suis*, Ide$_{Ssuis}$, leads to protection against a highly virulent serotype 9 strain. Vaccine X 2019;3. https://doi.org/10.1016/j.jvacx.2019.100046.
