# Supplementary material for: Immunogenicities of vaccines including the immunoglobulin M-degrading enzyme of Streptococcus suis, rIdeSsuis, and protective efficacy against serotype 14 in piglets
Source: Vaccine X. 2024 Nov 21;21:100590. doi: 10.1016/j.jvacx.2024.100590 (PMC11629322; doi:10.1016/j.jvacx.2024.100590)
Supplement: Supplementary file 2 — Table S2: Primary data of hemolysis assays [file mmc2.pdf]

Supplementary data file 2

Table S2: Primary data of hemolysis assays

Number of experiment	% of hemolysis																	
	rIde$_{Ssuis}$-vaccinated									Placebo-treated								
1st	84.3	11.6	57.3	4.6	93.3	92.5	70.9	7.3	96.5	9.7	9.3	10.8	6.0	8.9	6.8	6.3	7.9	8.3
2nd	30.7	9.4	76.7	2.4	80.3	89.5	72.2	6.9	82.9	10.7	11.0	9.4	6.1	10.1	7.0	4.6	5.5	5.9
3rd	35.1	8.5	52.7	2.8	88.2	92.5	72.0	7.1	92.5	8.0	11.7	10.7	8.6	10.7	6.1	5.7	7.1	6.2
mean	50.0	9.8	62.3	3.3	87.3	91.5	71.7	7.1	90.6	9.5	10.7	10.3	6.9	9.9	6.7	5.6	6.8	6.8
S.D.	29.8	1.6	12.8	1.2	6.5	1.8	0.7	0.2	7.0	1.4	1.2	0.7	1.4	0.9	0.5	0.9	1.2	1.3
