# Supplementary material for: Immunogenicities of vaccines including the immunoglobulin M-degrading enzyme of Streptococcus suis, rIdeSsuis, and protective efficacy against serotype 14 in piglets
Source: Vaccine X. 2024 Nov 21;21:100590. doi: 10.1016/j.jvacx.2024.100590 (PMC11629322; doi:10.1016/j.jvacx.2024.100590)
Supplement: Supplementary file 3 — Fig. S1 [file mmc3.pdf]

Supplementary data file 3

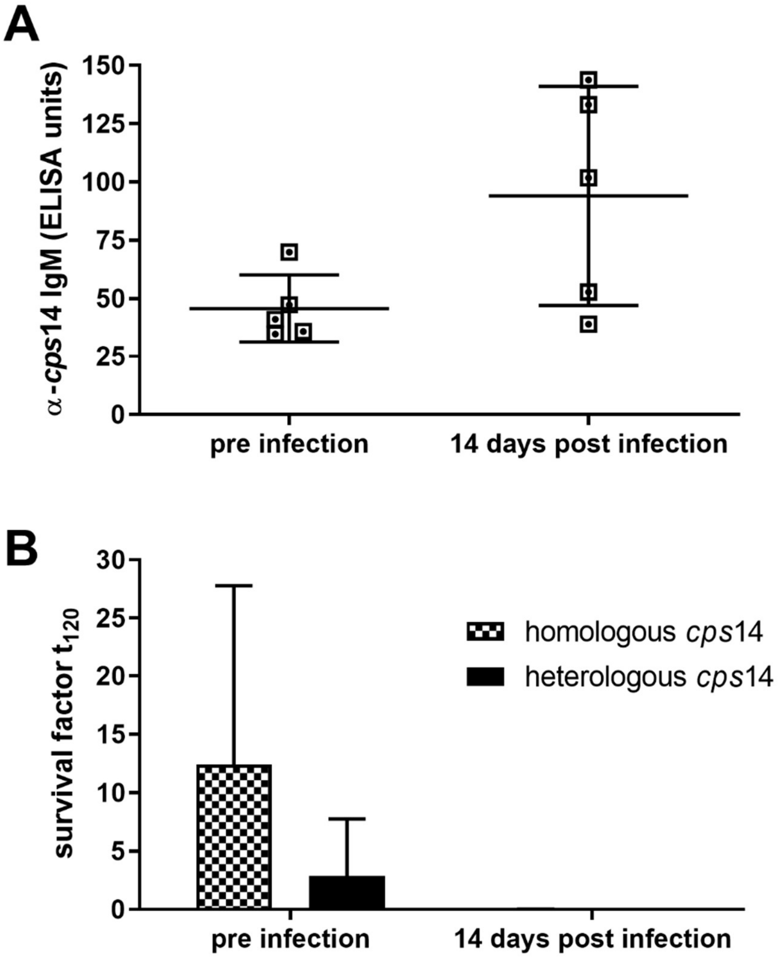

Fig. S1: Fourteen days post experimental c*ps*14 challenge, serum IgM binding to the surface of the streptococci is increased (A) and a homologous and a heterologous *cps*14 *S. suis* strain in is killed in reconstituted blood (B)

A IgM binding to formaldehyde-inactivated streptococci (*cps*14 strain V3117/2) was determined in serum samples collected prior to infection and 14 days post *cps*14 infection of five placebo-treated survivors in an ELISA.

B The same serum samples as in A were used to reconstitute 200 µl blood consisting of 100 µl serum and 100 µl porcine blood cells. Reconstituted blood samples were

subsequently inoculated with 2.4×10^5 CFU of either *cps*14 strain V3117/2 (homologous *cps*14 strain) or TW 078/11 (heterologous *cps*14 strain). The survival factor shows the ratio of CFU at 120 min to CFU at time point zero. Bars and error bars represent mean values and standard deviations, respectively.

A+B: No significant differences were determined using Wilcoxon test (**B**: only comparison of time points).
